# The Tie-2 agonist PMC-403 inhibits endothelial damage induced by e-cigarette vapor or LPS

**DOI:** 10.1186/s12931-026-03928-4

**Published:** 2026-09-26

**Authors:** Thorben Pape, Oleg Pak, Theresa Graalmann, Christine Ehlers, Janik Deutscher, David Spiegelberg, Paul Horn, Klaus Stahl, Marius M. Hoeper, Sascha David, Natascha Sommer, Benjamin Seeliger

**Affiliations:** 1 Department of Respiratory Medicine and Infectious Diseases, Hannover Medical School, Carl-Neuberg-Str.1, Hannover, 30625 Germany; 2 Biomedical Research in Endstage and Obstructive Lung Disease Hannover (BREATH), German Center for Lung Research (DZL), Hannover, Germany; 3 PRACTIS Clinician Scientist Program, Dean’s Office for Academic Career Development, Hannover Medical School, Hannover, Germany; 4 Excellence Cluster Cardio-Pulmonary Institute, University of Giessen and Marburg Lung Center (UGMLC), German Center for Lung Research (DZL), Institute for Lung Health (ILH), Justus Liebig University Giessen, Giessen, Germany; 5 Junior Research Group Translational Immunology, TWINCORE, Centre for Experimental and Clinical Infection Research, Helmholtz Centre for Infection Research and the Hannover Medical School, Hannover, Germany; 6 Department of Rheumatology and Immunology, Hannover Medical School, Hannover, Germany; 7 Department of Hepatology and Gastroenterology, Campus Virchow-Klinikum (CVK) and Campus Charité Mitte (CCM), Charité - Universitätsmedizin Berlin, Berlin, Germany; 8 Berlin Institute of Health at Charité – Universitätsmedizin Berlin, BIH Biomedical Innovation Academy, BIH Charité Digital Clinician Scientist Program, Berlin, Germany; 9 Department of Gastroenterology, Hepatology, Infectious Diseases and Endocrinology, Hannover Medical School, Hannover, Germany; 10 Institute of Intensive Care Medicine, University Hospital Zürich and University of Zurich, Zurich, Switzerland; 11 Department of Nephrology and Hypertension, Hannover Medical School, Hannover, Germany

**Keywords:** Systemic inflammation, ARDS, Capillary leakage, ECVE, ECV

## Abstract

**Background:**

The use of e-cigarettes is currently increasing despite potential harmful effects of e-cigarettes. Acute e-cigarette exposure may cause endothelial damage thereby contributing to acute lung injury or vascular dysfunction after chronic use. These effects may be enhanced by concomitant inflammatory stimuli.

We investigated the acute effect of e-cigarette vapor (ECV) with or without concomitant lipopolysaccharide (LPS) stimulation on endothelial cell function *in vitro* and on vascular permeability in isolated lungs. Further potentially protective effects of the Tie2 agonist PMC-403 were studied.

**Methods:**

Human umbilical cord endothelial cells (HUVECs) were cultured with different types of ECV extracts (ECVE) and/or LPS, and endothelial activation was determined *in vitro* by measuring secreted protein and mRNA levels of angiopoietin-2 (Angpt-2) and soluble Tie2 (sTie2). Barrier dysfunction was analyzed by continuous transendothelial electrical resistance (TER) measurements using electric cell-substrate impendence sensing (ECIS). An *ex vivo* isolated mouse lung model was used to analyze endothelial permeability and edema formation upon intratracheal ECV exposure. The Tie2 agonist PMC-403 was applied *in vitro* and *ex vivo* and its effect on endothelial permeability was determined.

**Results:**

Angpt-2 secretion and transcription were significantly increased in HUVECs after 24h stimulation with nicotine-containing ECVE (ECVE_nic_), but not nicotine-free ECVE. Preincubation with ECVE_nic_ significantly enhanced the LPS-induced increase of Angpt-2 secretion. On a functional level, ECVE_nic_ exposure resulted in greater susceptibility to an LPS-induced increase in endothelial barrier permeability. In isolated, ventilated and perfused mouse lungs intratracheal nicotine-containing ECV application (ECV_nic_) or LPS application via the perfusate increased endothelial permeability and lung edema, however, combination of ECV_nic_ and LPS did not have additive effects. A novel synthetic Tie2 agonist PMC-403 decreased both the LPS-induced loss of endothelial barrier function *in vitro* and the ECV_nic_- or LPS-induced endothelial barrier dysfunction *ex vivo*.

**Conclusions:**

ECV_nic_ exposure increased endothelial permeability and lung edema formation in an angiopoietin/Tie2 dependent manner *ex vivo*, and susceptibility to LPS stimulation *in vitro*. We identified the Tie2 activating anti-permeability agent PMC-403 as a potential therapeutic agent of acute lung injury in this context.

**Supplementary Information:**

The online version contains supplementary material available at https://doi.org/10.1186/s12931-026-03928-4.

## Background

The use of e-cigarettes is steadily increasing globally [1, 2]. In 2019, up to 6.6% of the European population reported active use of e-cigarettes [3]. Amongst e-cigarette users up to 55% report using e-cigarettes on a daily basis and 48% report daily use of nicotine-containing e-cigarette liquid, rendering it a public health concern [2, 4, 5]. The prevalence is particularly high in adolescents and young adults, males and people who smoke or have smoked conventional cigarettes [2, 4, 6]. In general, conventional smokers often attempt to quit smoking by using e-cigarettes which, however, is not recommended by medical associations [2, 7, 8]. Previously, e-cigarette use has been reported to cause acute lung injury (E-cigarette, or vaping, product use-associated lung injury, EVALI) with the need for invasive ventilation in up to 26% of patients [1].

An outbreak of EVALI cases with 2600 reported cases and 68 deaths has been described in 2019 and 2020 in the US [1, 9, 10]. Unfortunately, no specific treatment exists and EVALI is mainly treated by removing the injurious substance and providing supportive care [1]. Although vitamin E additives in e-cigarettes were identified as a key trigger for EVALI, the exact pathomechanism and pathophysiology remain unknown [1, 11].

Clinically, EVALI presents with extensive pulmonary infiltrates and increased endothelial permeability leading to capillary leakage [1, 12]. In non-e-cigarette induced acute respiratory distress syndrome (ARDS) and sepsis, the angiopoietin (Angpt)-Tie2 system is an important molecular modulator of endothelial permeability [13–15]. Tie2 is an endothelial transmembrane tyrosine kinase. Under healthy conditions, endothelial Tie2 is constitutively activated by binding of its circulating ligand angiopoietin-1 (Angpt-1) leading to its phosphorylation and thereby activation of downstream signaling pathways protecting endothelial integrity and function [13–15]. Angiopoietin-2 (Angpt-2) is stored in Weibel-Palade bodies of endothelial cells and released upon injurious stimulation (e.g. sepsis) [15]. Angpt-2 functions as an inhibitor of Tie2 and leads to its deactivation, consecutively leading to increased endothelial permeability and endothelial-mediated inflammation [13–15]. Increased Angpt-2 levels in the blood are associated with higher morbidity and mortality in patients with sepsis [16]. However, so far, no therapy targeting this endothelium-destabilizing system has been approved.

In this study we investigated the effects of nicotine-free or nicotine-containing e-cigarette vapor extract (ECVE) on the integrity of the endothelial barrier function in the presence or absence of a concomitant inflammatory stimulus (i.e. lipopolysaccharide, LPS), and the role of the Angpt/Tie2 system. Moreover, we hypothesized that a novel synthetic Tie2 agonist, PMC-403, can reduce deleterious inhibitory effects of Angpt-2 on Tie2 and thereby attenuate capillary leak in vitro and ex vivo.

## Materials and methods

### Antibodies and reagents

All reagents were purchased from Sigma Aldrich (St. Louis, MO) unless otherwise specified. Antibodies against Akt (19 ng/ml) (11E7, Cell Signaling Technology, Cambridge, UK), pAkt (10 ng/ml) (Ser473, Cell Signaling Technology, Cambridge, UK) and GAPDH (200 ng/ml) (FL-335, Santa Cruz Biotechnology, CA) were utilized. As secondary antibodies goat anti-rabbit IgG-HRP (160 ng/ml) (Santa Cruz Biotechnology, CA) and donkey anti-goat IgG-HRP (160 ng/ml) (Santa Cruz Biotechnology, CA) were used.

### Cell culture studies

Informed consent was obtained from all umbilical cord donors and the protocol was approved by the ethics committee of Hannover Medical School (No. 1303 - 2012).

In accordance with governmental and institutional guidelines, the umbilical vein was cannulated and flushed with phosphate-buffered saline (PBS, Thermo Fisher Scientific, Waltham, MA) to remove residual blood. The vein was subsequently filled with collagenase solution (Biochrom, Berlin, Germany) and incubated at 37 °C for 20–30 min. Detached endothelial cells were collected into PBS supplemented with heat-inactivated fetal bovine serum (FBS, Thermo Fisher Scientific, Waltham, MA), centrifuged, and resuspended in endothelial growth medium (EBM-2, Lonza, Basel, Switzerland) for subsequent culture. Further details on HUVEC culture have been described previously [17, 18].

As an additional endothelial cell type, primary human pulmonary microvascular endothelial cells (HPMECs; PromoCell, Heidelberg, Germany; Cat. No. C-12281) were used and cultured in endothelial cell growth medium MV2 (PromoCell; Cat. No. C-22022).

Unless specified otherwise, cells were stimulated with 100 ng/ml lipopolysaccharide (LPS) from E. coli serotype O111:B4 (L4130, Sigma-Aldrich, St. Louis, MO) or vehicle (EBM-2), 100 ng/ml lipopolysaccharide binding protein (LBP) (870-LP-025, R&D Systems, Minneapolis, MN), 100 ng/ml CD14 (SRP3149, Sigma–Aldrich, St. Louis, MO), 500 ng/ml Angpt-1 (923-AN/CF, R&D Systems, Minneapolis, MN), electronic cigarette vapor extract (ECVE, see below) and/or different concentrations (e.g. 0.3, 1, 3–10 µg/ml) of the Tie2 agonist PMC-403 (PharmAbcine, Daejeon, Republic of Korea).

### Enzyme-linked Immunosorbent Assay (ELISA)

ELISA was performed from endothelial cell culture supernatants and endothelial cell lysates with specific commercial human ELISA kits for Angiopoietin-2 (Angpt-2) (DY623, R&D Systems, Minneapolis, MN). All additionally needed reagents were purchased from R&D Systems except for Normal Mouse Serum (NMS) (Jackson ImmunoResearch Laboratories, Westgrove, PA) and Bovine Serum Albumin (BSA) (Sigma-Aldrich, St. Louis, MO).

### RNA isolation and quantitative PCR

RNA from cultured HUVECs was isolated using the RNeasy MicroKit (Qiagen, Hilden, Germany) according to the manufacturer’s instructions. With the transcriptor First Stand cDNA Synthesis kit 0.5 µg of total extracted RNA was reverse transcribed to cDNA (Roche Diagnostics, Rotkreuz, Switzerland). SYBR Green real-time PCR was performed using a LightCycler 480II (Roche, Basel, Switzerland) and LightCycler 480 SYBR Green I Master (Roche, Basel, Switzerland). Primers were synthesized by Eurofins Genomics (Ebersberg, Germany), prepared as 100 µM stock solutions and diluted to 10 µM working solutions. Forward and reverse primers were used at a final concentration of 300 nM. Primers were designed using NCBI Primer-BLAST, preferentially spanning exon–exon junctions where possible and checked against the RefSeq transcriptome for a single predicted amplicon. Specificity was confirmed by single-peak melt-curve analysis, and no-template controls (NTC) were included on each plate. The following primers were used: human ß-Actin (fv: CTG GAA CGG TGA AGG TGA CA, rev: AGT CCT CGG CCA CAT TGT G), human Angpt-2 (fv: GCC GCT CGA ATA CGA TGA CT, rev: GCT TCA TTA GCC ACT GAG TGT TGT), human Tie-2 (fv: CAG TAC GTG GTC CGA GCT AGA GT, rev: TAA GGG TCC AAG CAG TGA GAT CT), human ICAM-1 (fv: CGT GGG GAG AAG GAG CTG AA, rev: CAG TGC GGC ACG AGA AAT TG), human VCAM (fv: TGA CAA GTC CCC ATC GTT GA, rev: ACC TCG CGA CGG CAT AAT T). For each sample we performed triplicates of RT-qPCR reactions and averages of the cycle values were used for quantification.

### Electronic Cigarette Vapor Extract (ECVE) production *in vitro*

**Fig. 1:**
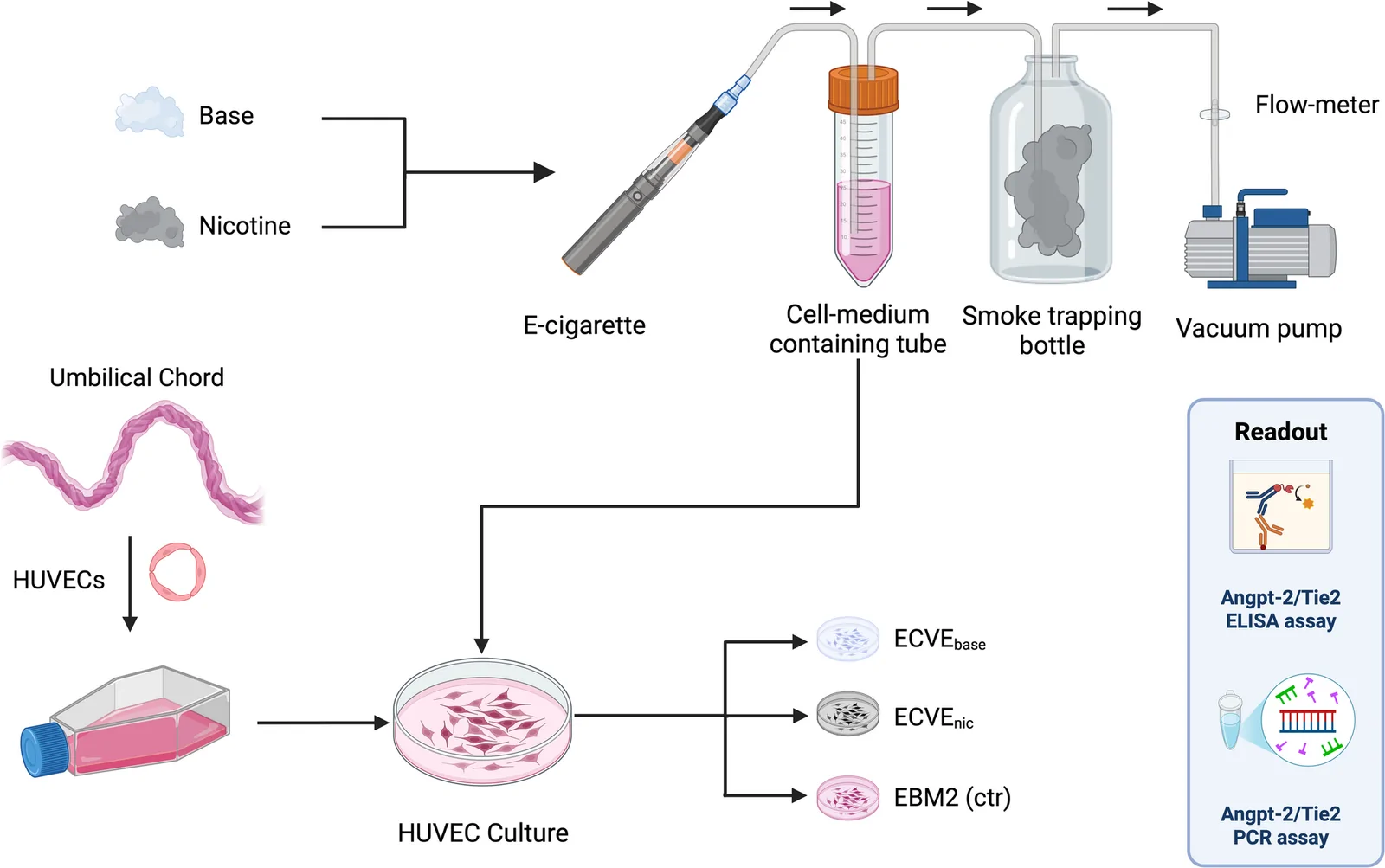
The cartridge of the e-cigarette was filled with either base or nicotine. The vape was caught by tubes and directed to a falcon filled with endothelial growth medium. The closed system was attached to a smoke trapping bottle which was ultimately attached to a vacuum pump, thereby imitating a standardized vaping process. The resulting medium was then filtered through a 0.20 µm filter, considered 100% and used for further experiments. Created in BioRender. Stahl, K. (2026) https://BioRender.com/jepgrde. Abbreviations: Angpt-2 – Angiopoietin-2, ECVE – e-cigarette vapor extract, HUVEC – Human umbilical vein endothelial cells

To imitate e-cigarette smoking, we set up a novel vaporizing construction to produce e-cigarette vapor extract (ECVE). An e-cigarette (Joyetech eGo-C, Riccardo Retail GmbH, Neubrandenburg, Germany) filled with e-liquid (VG: PG based) (Riccardo Retail GmbH, Neubrandenburg, Germany) with a glycol/glycerol/water ratio of 55/35/10% (base), which is widely applied by customers (in Germany) [19] and in line with established approaches in the literature [20–22], with (18 mg/ml) and without nicotine was bubbled through a falcon with 10 ml of endothelial growth medium (EBM-2, Lonza, Basel, Switzerland) as previously described [19]. The closed system was attached to a smoke trapping bottle and a vacuum pump to retract smoke from the falcon with cell medium (Fig. 1) and imitate a normal vaping process.

This suspension from the falcon was then filtered through a 0.20 μm pore filter. The filtered ECVE stock solution was operationally defined as 100% ECVE, indicating that it was used without further dilution and not that saturation of nicotine or other vapor constituents had been reached. ECVE was generated under identical standardized conditions for each experiment (e-cigarette: 30 W, TLR1, 0.61 Ohm, 0.72 A, 4.28 V; puffing conditions: 15 puffs with a duration of 4 s following 20 s without puffing) and used immediately after preparation without storage. ECVE_base_ (base without nicotine), ECVE_nic_ (base with nicotine) and only growth endothelial medium (EBM-2) as negative control were used respectively. Potential variation between individual ECVE preparations was minimized by these standardized preparation conditions. However, potential batch-to-batch differences in individual ECVE constituents were not directly quantified.

Unless stated otherwise, undiluted ECVE (100%) (ECVE_nic_) was used. ECVE_base_ is defined by non-nicotine containing e-liquid.

### E-cigarette vapor application in isolated ventilated and perfused mouse lungs

All mouse experiments were approved by the local authorities at the Justus Liebig University Giessen and conducted in accordance with institutional and governmental guidelines according to the German Animal Welfare Law (Regierungspraesidium Giessen, Germany). Mice were heparinized (2,500 U/kg BW) and deeply anesthetized with ketamine/xylazine (100/20 mg/kg BW, i.p.), followed by tracheostomy, intubation, and normoxic ventilation (21% O_2_, 5.3% CO_2_, balanced with N_2_; Minivent 845 (Hugo Sachs Elektronik March-Hugstetten, Germany); tidal volume 10 µl/g BW, 90 breaths/min, PEEP 3 cm H_2_O). After thoracotomy, the lung–heart block was excised and perfused via the pulmonary artery with Krebs-Henseleit buffer.

Nicotine-containing e-cigarette vapor (ECV_nic_) was generated using an eVic Primo Mini (Joyetech^®^, 0.5 Ω, 30 W) and applied via the ventilator system (4 times 5 s puffs, 150 applications over 5 min). Control preparations received normoxic gas. LPS (6 µg/ml), the Tie2 agonist PMC-403 (10 µg/ml) or vehicle, or their combination were added to the perfusate 5 min before ECV_nic_ exposure.

After ECV_nic_ inhalation, transvascular fluid filtration (endothelial permeability) was assessed under hydrostatic stress by increasing venous pressure from 2 to 12 cm H₂O for 8 min (“pressure challenge”), calculating the capillary filtration coefficient (K_fc_) as previously described [23]. Pulmonary edema formation was quantified by lung weight gain and the wet-to-dry weight ratio.

A detailed description is provided in the Supplemental Material (Supplemental Data 1).

### Transendothelial Electrical Resistance (TER)

Special cell culture plates (ibidi, 8W10E) were coated for 1 h at 37 °C with 200 µl collagen type I (dilution 1:50, C8919, Sigma Aldrich, St. Louis, MO). All wells were coated using the same collagen solution, concentration, volume, incubation time, and standardized manual procedure to minimize coating-related variability. The collagen solution was then carefully removed and 400 µl of cell suspension in growth medium was added. Cells were grown to confluency at 37 °C. Monolayer confluency was determined regarding manufacturer’s recommendation by electrical criteria (resistance > 1800 ohms and capacitance > 10 nF). Cells were either stimulated with ECVE medium and/or 100 ng/ml LPS accordingly. For some experiments, cells were pretreated with 10 µg/ml PMC-403 or vehicle 1 h prior to LPS stimulation. Medium was changed by carefully removing the medium and replacing it carefully to avoid disturbing the cell monolayer in these steps. Transendothelial electrical resistance (TER) was measured at different time points by an electric cell-substrate impedance sensing system (ECIS, Applied BioPhysics Inc.). Values were either plotted over time or as dot plots at time points of maximal response with mean and standard deviation (SD) as described before [24, 25]. Resistances of each condition at each time point were divided by its condition starting resistance to calculate normalized TER.

### MTT assay (“cell viability”)

Cell morphology and adherence were assessed by phase-contrast microscopy. The MTT assay is based on the cleavage of the tetrazolium salt, 3,(4,5-dimethylthiazol-2-yl)-2,5-diphenyl-tetrazolium bromide to formazan salt in living cells (MTT). The commercial MTT colorimetric cell viability kit IV (PK-CA707-30006-200, PromoCell, Heidelberg, Germany) was used according to manufacturer’s instructions followed by photospectrometric quantification at 570 nm.

### Immunoblotting

Cells were lysed by radioimmunoprecipitation buffer (including 1 mM Na3VO4, 50 mM NaF, protease inhibitors, Roche Diagnostics, Mannheim, Germany) after initial washing with PBS (Sigma-Aldrich, St. Louis, MO). Next, lysates were centrifuged for 15 min at 4 °C at 12,000 rpm and the supernatants were collected. The protein concentration of the supernatant was measured with Pierce BCA Protein Assay Kit (Thermo Scientific, Rockland, IL). SDS polyacrylamide gel electrophoresis was used to separate proteins. The proteins were then transferred to PVDF (polyvinylidene fluoride) membranes (Merck Millipore, Darmstadt, Germany) and incubated with appropriate antibodies (primary antibody overnight at 4 °C; secondary antibody for 1 h at room temperature). Next, the membrane was washed with TBST (20 mM Tris, 150 mM NaCl, 0.1% Tween20, Merck) and secondary antibodies conjugated to horseradish peroxidase (Santa Cruz Biotechnology, CA) were used to detect proteins. SuperSignal™ West Pico Chemiluminescent Substrate (Life Technologies, Carlsbad, CA) was used to enhance the chemiluminescence. Finally, the signal was detected with Versa Doc Imaging System Model 3000 (BioRad, Hercules, CA).

### Statistical analysis

Data were tested for Gaussian distribution with Shapiro-Wilk test. When data showed Gaussian distribution unpaired t-test with Welch’s correction for comparison of two independent groups or One-Way-ANOVA with Bonferroni post-hoc adjustment for multiple comparisons was used accordingly. When data did not show Gaussian distribution Mann-Whitney U test for comparison of two independent groups or Kruskal-Wallis test with Dunn’s post-hoc adjustment for multiple comparison were used. For comparison of different dependent groups at different time points mixed ANOVA with according post-hoc test was used (*anova*,* emmeans*) and generalized eta-squared was calculated. To analyze changes and differences over time (ECIS) mixed effects model (*lmer*) was used with according post-hoc analysis (*emmeans*, *emtrends*) and partial R^2^ (*r2glmm*) and coefficient ß were stated accordingly. Individual measurements were excluded in cases of technical failure or non-evaluable assay results. Consequently, sample sizes differed slightly between individual experimental groups. The exact sample sizes (n) are provided in the respective figure legends. Results were considered statistically significant with a *p* < 0.05. Dot plots are presented as mean ± SD. The R environment for statistical computing (Version 4.1.2, R Foundation for Statistical Computing, Vienna, Austria) was used for data analysis and graph generation.

## Results

### E-cigarette vapor extract increases the permeability-enhancing factor Angpt-2

**Fig. 2:**
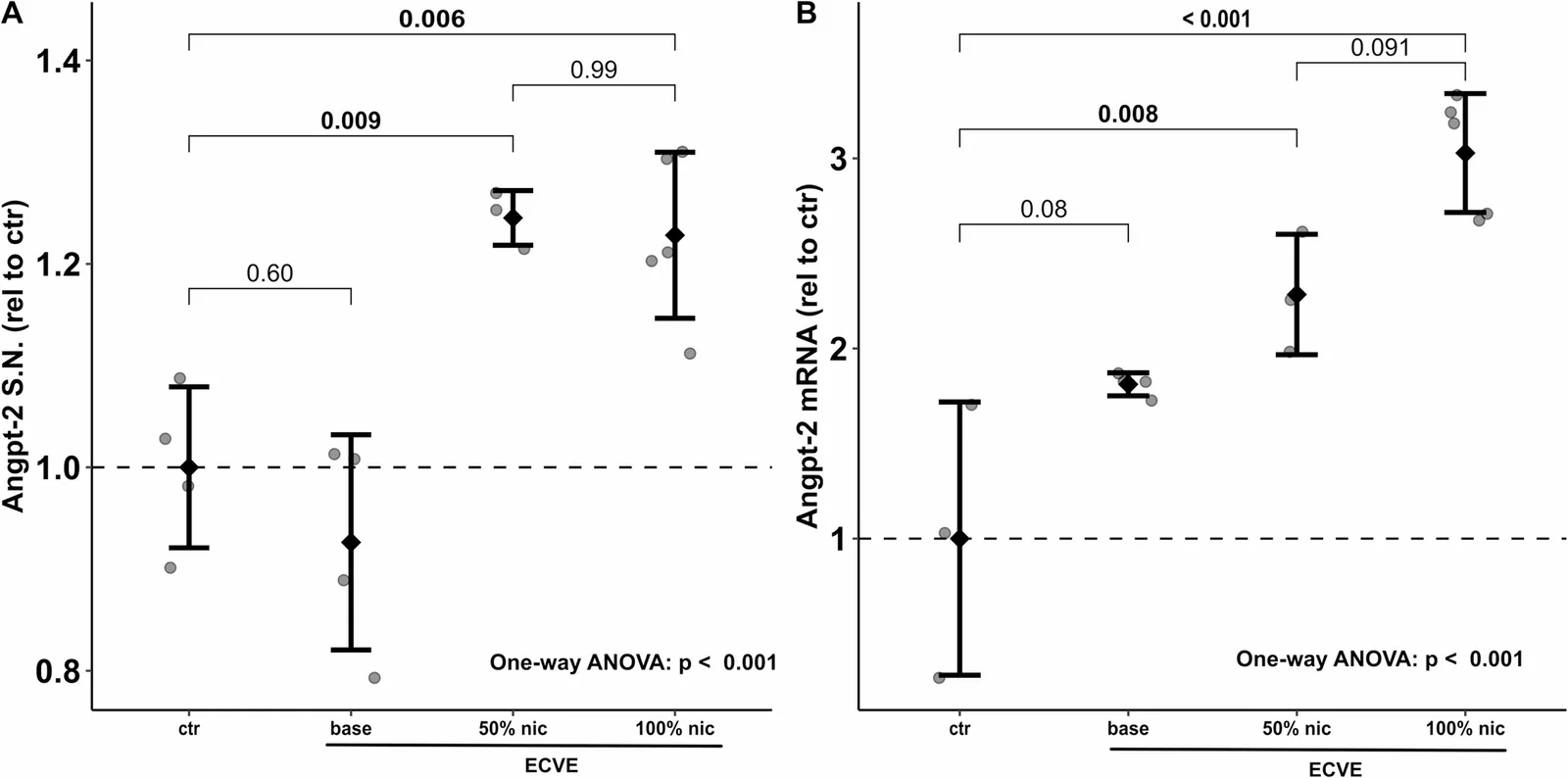
HUVECs were stimulated with either ECVE_base_ (*n*=4), ECVE_nic_ at different dilutions (*n*=3 for 50%, *n*=5 for 100%) or control (*n*=4 in A, *n*=3 in B) for 24 hours and (**A**) Angpt-2 concentrations in the supernatant were measured by ELISA. **B **Additionally, Angpt-2 mRNA transcription was measured by qPCR in HUVECs after stimulation for 24 hours. Results are shown as dot plots with mean ± SD. One-way ANOVA with Tukey’s post-hoc test was performed. *P*-values < 0.05 are considered significant. Abbreviations: Angpt-2 - Angiopoietin-2, ECVE – e-cigarette vapor extract, HUVECs – human umbilical vein endothelial cells, SD – standard deviation

First, we investigated the effect of ECVE on the Angpt/Tie2 system without additional inflammatory stimuli. HUVECs were stimulated for 24 h with different concentrations of ECVE_nic_ as well as ECVE_base_ and control (EBM-2, ctr). Stimulation with ECVE_nic_ led to increased Angpt-2 levels in the supernatant compared to control and base (ctr: 1.000 ± 0.079 vs. ECVE_nic(100%)_: 1.228 ± 0.082, *p* = 0.006), while nicotine-free ECVE (ECVE_base_) did not induce any significant differences compared to control (Fig. 2A). Moreover, Angpt-2 transcript levels were increased 24 h after ECVE stimulation depending on the dose of nicotine (ctr: 1.000 ± 0.719 vs. ECVE_nic(100%)_: 3.023 ± 0.313, *p* < 0.001) (Fig. 2B). ECVE_base_ only led to a numerically increased transcription of Angpt-2. Therefore, for further experiments we focused on ECVE_base_ vs. (100%) ECVE_nic_, as ECVE_base_ and control were not significantly different. The observed increase in Angpt-2 (that deactivates Tie2 phosphorylation and downstream targets such as cell adhesion molecules) was not associated with altered expression of Tie-2 transcripts in ECVE_base_ and ECVE_nic_ (Figure S1A) or the downstream cell adhesion molecules ICAM or VCAM (Figure S1B, C). Of note, cell toxicity upon ECVE_nic_ stimulation was excluded using an MTT cell viability assay. In summary, ECVE_nic_ increased Angpt-2 release and biosynthesis on the transcript level, while ECVE_base_ did not have any significant effects on Angpt-2 levels.

### ECVE increases the susceptibility to an inflammatory environment and capillary leakage *in vitro*

**Fig. 3:**
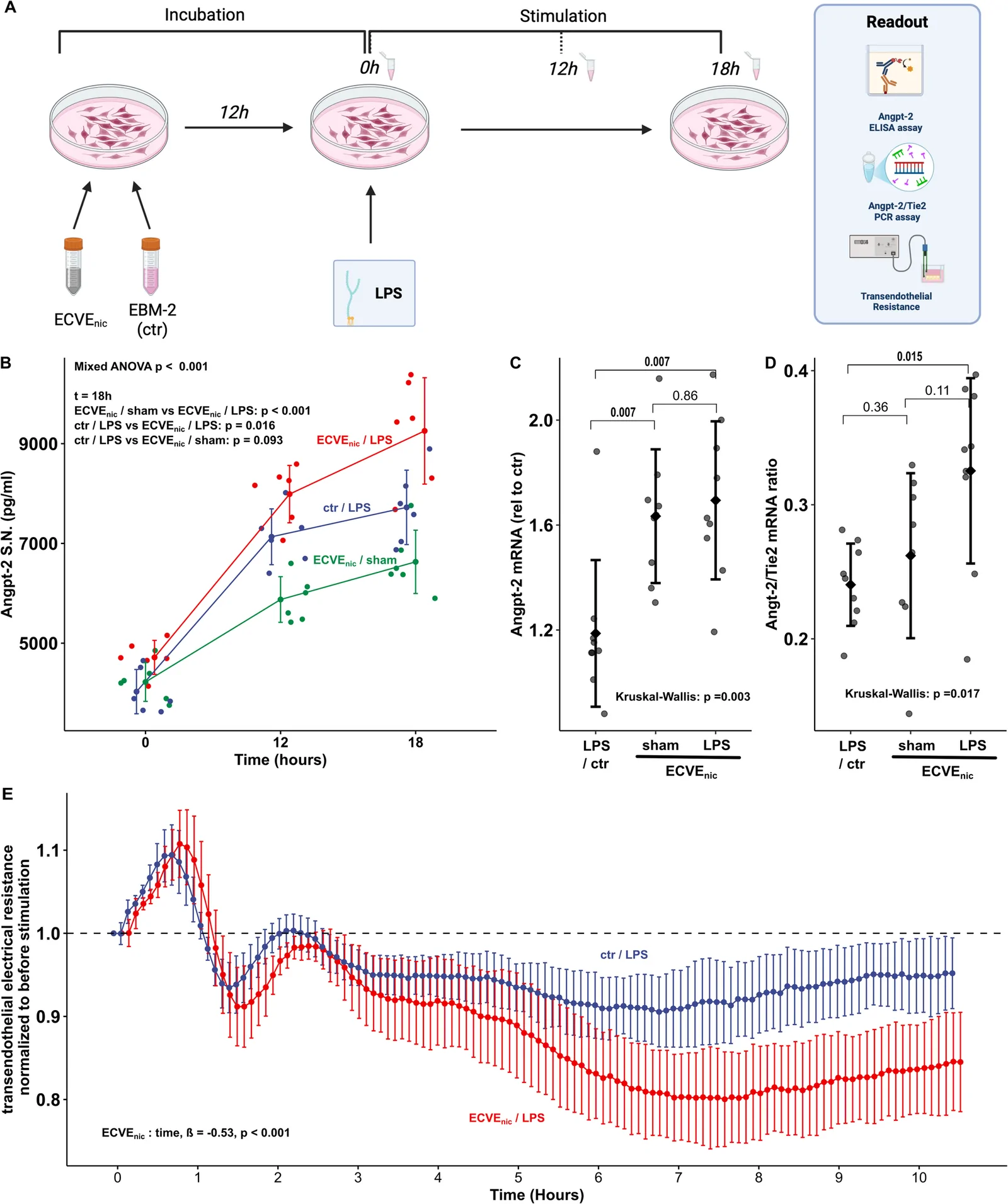
**A **HUVECs were stimulated with ECVE_nic_ or control (EBM-2) for 12 hours (incubation). Then, 100 ng/ml LPS with co-factors LBP and CD14 or co-factors alone (sham) were added and incubated for another 18 hours. Created in BioRender. Stahl, K. (2026) https://BioRender.com/nu4iwxz. **B **Angpt-2 concentrations in the supernatant were measured by ELISA when LPS was added as well as 12 and 18 hours after LPS or sham stimulation and are shown over time. Each group consisted of *n*=6. Mixed ANOVA with according post-tests were performed. **C **18 hours after stimulation with LPS or sham Angpt-2 mRNA transcription was measured by qPCR. Each group consisted of *n*=9. **D **Additionally, the Angpt-2/Tie2 mRNA ratio is shown after initial ECVE_nic_ incubation and 18 hours of LPS or sham stimulation. ECVE_nic_ / sham consisted of n=8, all other groups consisted of *n*=9. Groups were analyzed by Kruskal-Wallis test with post-hoc Dunn test. **E **TER was measured at different time points by an ECIS system. HUVECs were pre-incubated with ECVE_nic_ (*n*=7) or control (*n*=8) for 12 hours. Then half of the medium was changed and cells were stimulated with LPS. Results are shown as dot plots with mean ± SD. A linear mixed-effects model was used for analysis. *P*-values < 0.05 are considered significant. Abbreviations: Angpt-2 - Angiopoietin-2, ECIS - Electric cell-substrate impedance sensing, ECVE – e-cigarette vapor extract, HUVECs – human umbilical vein endothelial cells, LPS - lipopolysaccharide, SD – standard deviation, TER – transendothelial electrical resistance

As ECVE_nic_ increased Angpt-2 levels, we next analyzed its effect in an inflammatory environment. To this end, we pre-incubated HUVECs for 12 h with either ECVE_nic_ or control medium (EBM-2) and afterwards stimulated the cells with LPS together with the co-factors LBP and CD14 or the co-factors alone, which enhance LPS presentation to the Toll-like receptor (TLR)4 complex (downstream signaling) (Fig. 3A). If HUVECs were pre-incubated with ECVE_nic_ and subsequently stimulated with LPS, Angpt-2 levels were significantly higher compared to LPS or ECVE_nic_ alone (18 h, ctr/LPS: 7723 ± 746 pg/ml vs. ECVE_nic_/LPS: 9255 ± 1066 pg/ml, *p* = 0.016) showing an additive effect of ECVE_nic_ and LPS (Fig. 3B). The Angpt-2 transcript levels at 18 h were significantly increased after stimulation with ECVE_nic_ and LPS compared to stimulation with LPS alone (ctr/LPS: 1.187 ± 0.280 vs. ECVE_nic_/LPS: 1.694 ± 0.301, *p* = 0.004) (Fig. 3C). Of note, a variability can be seen between cells with one significantly lower and one significantly higher data point across all groups. Similarly, the Angpt-2/Tie2 ratio, as a more sensitive marker for Angpt-2 dependent Tie2 inhibition, showed the tendency for an additive effect of stimulation with LPS after pre-incubation with ECVE_nic_ (ctr/LPS: 0.240 ± 0.031 vs. ECVE_nic_/LPS: 0.325 ± 0.070, *p* = 0.015) (Fig. 3D).

To investigate functional effects of the additive effect of LPS stimulation after pre-incubation with ECVE_nic_ on the endothelial barrier function in vitro, ECIS-TER experiments were performed. TER was measured over time after LPS stimulation. The LPS-induced reduction in TER (i.e. increased permeability) was significantly more pronounced over time if HUVECs were pre-incubated with ECVE_nic_ (ß = -0.53, *p* < 0.001, e.g. normalized TER 7 h after LPS stimulation: ctr/LPS: 0.909 ± 0.046 vs. ECVE_nic_/LPS: 0.803 ± 0.052, *p* < 0.001, Fig. 3E).

In summary, pre-incubation with ECVE_nic_ increased susceptibility to an inflammatory stimulus reflected by higher Angpt-2 supernatant levels. On a functional level, ECVE_nic_ pre-disposed to a higher LPS-induced increase of endothelial permeability.

### The Tie2-agonist PMC-403 reduces susceptibility *in vitro*

**Fig. 4:**
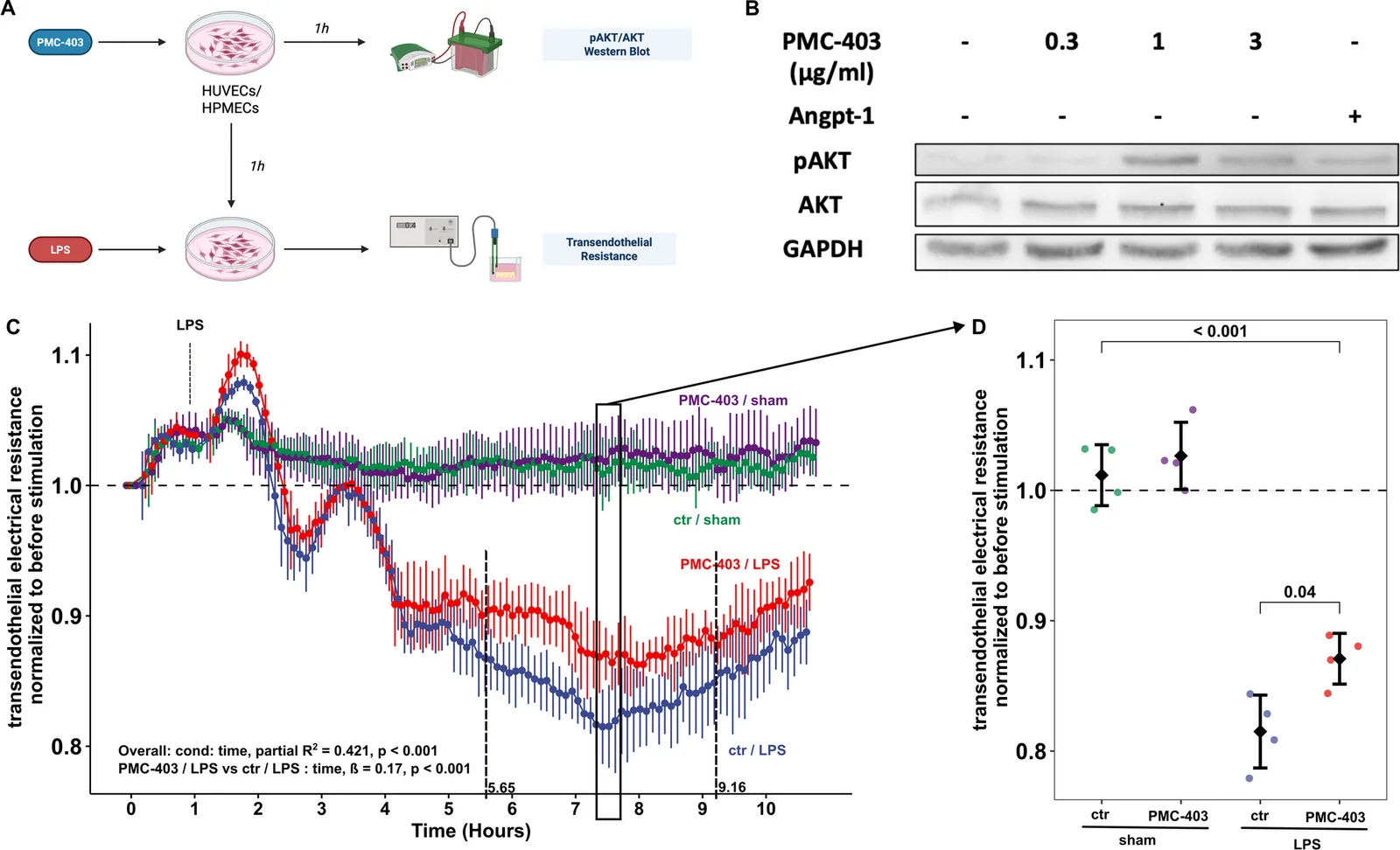
**A **A schematic setup is shown for stimulation with the Tie2 agonist PMC-403 and its effects on HUVECs and HPMECs, accordingly. Created in BioRender. Stahl, K. (2026) https://BioRender.com/yc7s3fj. **B **HUVECs were stimulated with different concentrations of the Tie2 agonist PMC-403 or 500 ng/ml Angpt-1 for 1 hour. A western blot was performed and pAKT and AKT were analyzed. **C **HPMECs were incubated with 1 µg/ml PMC-403 for 1 hour and then stimulated with LPS. TER was measured by ECIS over time and group differences over time were analyzed by a linear mixed-effects model accordingly. **D **Group differences are shown 6.5 hours after LPS stimulation. Results are shown as dot plots with mean ± SD and each group consisted of *n*=4. *P*-values < 0.05 are considered significant. Abbreviations: Angpt-1 - Angiopoietin-1, ECIS - Electric cell-substrate impedance sensing, HPMECs - human pulmonary microvascular endothelial cells, HUVECs – human umbilical vein endothelial cells, LPS - lipopolysaccharide, SD – standard deviation, TER – transendothelial electrical resistance

To investigate the effect of PMC-403 on Tie2 activation, we stimulated HUVECs with different concentrations of PMC-403 or 500 ng/ml Angpt-1 (stimulation of Tie2) for 1 h. As AKT is phosphorylated as part of the PI3K downstream signaling after Tie2 activation, we analyzed the levels of phosphorylated AKT (pAKT) (Fig. 4A, B). PMC-403 activated Tie2 downstream signaling (Fig. 4B). PMC-403 did not have any significant effects on the Angpt-2 release or transcription as well as the TER in HUVECs (Figure S2). Next, we wanted to characterize PMC-403 by well-known stimuli (LPS) in vitro as a proof-of-concept before testing its potential in an ex vivo setting. We analyzed the effect of PMC-403 on human pulmonary microvascular endothelial cells (HPMECs) to see if PMC-403 has a protective effect on pulmonary vascular leakage. Therefore, we pre-incubated HPMECs for 1 h with PMC-403 and subsequently added LPS. TER was measured over time, and PMC-403 led to a significant trend difference in an inflammatory environment from 5.7 to 9.2 h (PMC-403 / LPS vs. ctr / LPS: ß = 0.17, *p* < 0.001) (Fig. 4C) and a significant difference at the maximal barrier breakdown before established natural barrier rescue in vitro approx. 6.5 h after LPS stimulation (i.e. normalized TER 6.5 h after LPS stimulation: ctr/LPS: 0.815 ± 0.028 vs. PMC-403/LPS: 0.871 ± 0.019, *p* = 0.035) (Fig. 4D).

### PMC-403 rescues endothelial barrier dysfunction in isolated lungs

**Fig. 5:**
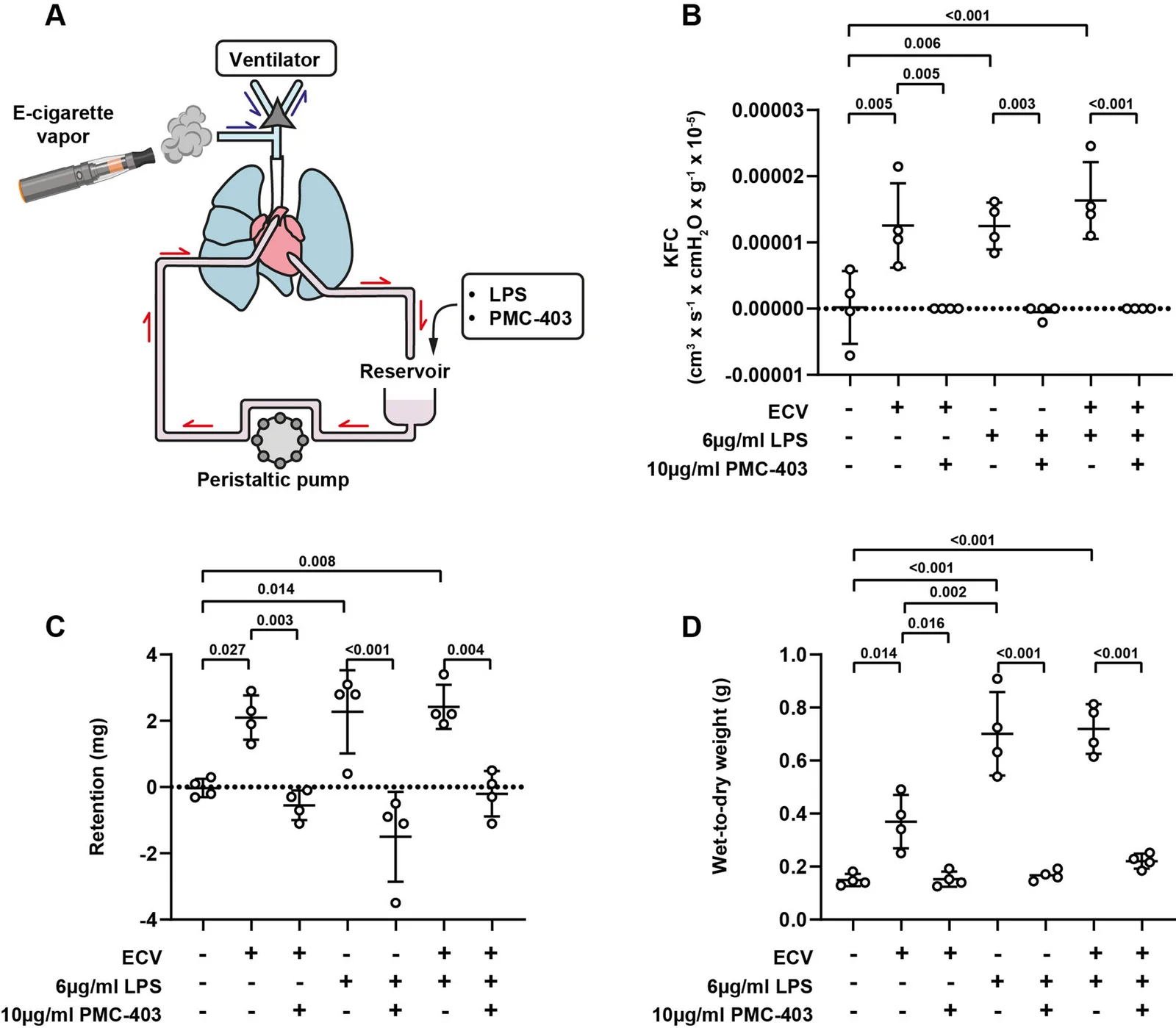
**A **Schematic of the experimental setup using isolated, perfused, and ventilated mouse lungs. Nicotine-containing e-cigarette vapor (ECV_nic_) was administered intratracheally. Lungs were ventilated via the trachea and perfused through the pulmonary artery, with the perfusate recirculated via a catheter placed in the left atrium. Tie2-agonist PMC-403 was added to the perfusate at concentrations of 6 µg/ml and 10 µg/ml. Effects of PMC-403 on (**B**) capillary filtration coefficient (K_fc_), (**C**) fluid retention, and (**D**) wet-to-dry lung weight ratio are shown. All groups consisted of *n*=4. Statistical analysis was performed using one-way ANOVA with according post-hoc tests. Data are presented as mean ± SD. *P*-values < 0.05 are considered significant. Abbreviations: ECV – e-cigarette vapor, K_fc_ – capillary filtration coefficient, SD – standard deviation

To further investigate the effect of the Tie2-agonist PMC-403 on endothelial barrier function, we used ex vivo isolated, ventilated, and perfused mouse lungs (Fig. 5A). The dose of PMC-403 was determined based on our in vitro experiments. We found in our previous work that the 10-fold concentration of substances used in vitro are effective in the isolated lung model (ex vivo) [26]. Intratracheal application of nicotine-containing e-cigarette vapor ECV (ECV_nic_) increased endothelial permeability (K_fc_: ctr: 1.838 × 10^− 7^ ± 5.494 × 10^− 6^ vs. ECV: 1.254 × 10^− 5^ ± 6.371 × 10^− 6^, *p* = 0.005, Fig. 5B), edema formation (Retention: ctr: -0.027 ± 0.278 vs. ECV: 2.100 ± 0.673, *p* = 0.027, Fig. 5C), and the wet-to-dry weight ratio (ctr: 0.149 ± 0.022 vs. ECV: 0.369 ± 0.101, Fig. 5D). Perfusion with 6 µg/ml LPS, as found appropriate in our previous work [27], increased endothelial permeability, edema formation, and wet-to-dry ratio (Fig. 5B–D), but did not further enhance the effects of ECV_nic_ inhalation. However, LPS induced a stronger increase in the wet-to-dry ratio, suggesting greater edema formation compared to ECV alone. Notably, treatment with the Tie2-agonist PMC-403 at 10 µg/ml effectively inhibited barrier disruption induced by ECV_nic_, LPS and the combination of ECV_nic_ and LPS (Fig. 5B–D).

## Discussion

In this study, we investigated the effect of ECVE on the Angpt/Tie2 system in vitro and found an increase in the permeability-inducing factor Angpt-2 by ECVE_nic_. Furthermore, ECVE_nic_ increased susceptibility to an inflammatory stimulus, i.e. LPS. Notably, e-cigarette vapor increased endothelial barrier dysfunction in vitro and ex vivo*.* The Tie2 agonist PMC-403 reduced endothelial permeability in inflammatory environments in vitro and ex vivo. These findings align with clinical observations in EVALI, which presents with diffuse pulmonary infiltrates, hypoxemia, and radiological features that are consistent with acute injury to the alveolo-capillary barrier [12, 28] and earlier findings that nicotine-containing e-cigarettes may induce pulmonary inflammation [29, 30].

The Angpt/Tie2 pathway is a key regulator of endothelial quiescence and permeability. The circulating Tie2-activating ligand Angpt-1 promotes anti-permeability and anti-inflammation effects, whereas Angpt-2, stored in Weibel-Palade bodies, is released upon inflammation and inhibits the Tie2 receptor leading to inflammation and increased permeability [31–33]. In sepsis, elevated Angpt-2 is associated with endothelial activation and worse outcomes, supporting its role as both a biomarker and an effector of vascular leakage [16, 32]. Our data show that Angpt-2 release is increased at baseline by ECVE_nic_ which may contribute to EVALI. Moreover, ECVE_nic_ augmented the susceptibility to LPS (synergistic effect). Notably, the transcriptional response showed a different pattern as combined ECVE_nic_/LPS exposure was increased compared with LPS alone but not compared with ECVE_nic_ alone. This difference between Angpt-2 release and transcription is not necessarily contradictory, as Angpt-2 is stored in Weibel-Palade bodies and can be released independently of simultaneous transcriptional changes [31–33]. These findings support involvement of Angpt/Tie2 dysregulation but also suggest that additional endothelial signaling mechanisms may contribute to the observed barrier dysfunction. Determined by TER measurements, ECVE_nic_ increased endothelial permeability compared to LPS alone. The ability to determine the independent contribution of ECVE_nic_ and to formally assess an additive or synergistic interaction with LPS in this functional assay is limited as an ECVE_nic_ control is missing. In line, Sussan et al. have shown that e-cigarette exposure impairs innate host defense and increases susceptibility to pathogen-associated stimuli [30]. According to previous findings that show a more pronounced effect of nicotine-containing ECVE compared to nicotine-free ECVE, we did not find any effect of nicotine-free ECVE on Angpt-2 levels [19].

Besides nicotine-dependent mechanisms, additives in e-cigarettes play a central role for development of EVALI. Previous investigations identified vitamin E acetate (VEA) in the bronchoalveolar lavage fluid of EVALI patients and found toxic pyrolysis products (e.g., ketene) as plausible drivers of injury [11, 34, 35]. Although, data on the effect of VEA on the Angpt/Tie2 system exist, these focus on cancer treatment and angiogenesis research. Suppression of the Angpt-1 has been found after treatment with vitamin E underlining its antiangiogenic properties [36, 37]. Nevertheless, no effect on Angpt-2 was observed in these studies [37]. On the contrary, Hama et al. show that α-Tocopheryl succinate inhibits Angpt-2 expression leading to stabilization of vascular structure [38]. These data suggest a complex role of the Angpt/Tie2 system with potentially different roles in angiogenesis and inflammation. To our knowledge, no data on the effect of VEA concerning pulmonary arterial permeability in inflammation exist and need further investigation in the context of VEA as a trigger for EVALI. Of note, the current literature does not provide clear evidence linking individual e-cigarette vapor constituents to Angpt-2 regulation. Existing studies primarily address endothelial dysfunction, oxidative stress, and barrier disruption, rather than Angpt-2-specific mechanisms. While nicotine and reactive aldehydes are established modulators of endothelial signaling [19, 39], attribution of Angpt-2 effects to a single compound is currently not supported by available data.

In contrast to the in vitro model, ECV_nic_ did not consistently potentiate the effect of LPS on pulmonary barrier dysfunction in the isolated mouse lung model. This is most likely due to differences in the methodological approaches by applying ECV and LPS simultaneously in the ex vivo model while having pre-incubated in the in vitro setting. Therefore, we can only speculate that the synergistic effects of ECV and LPS observed in vitro may require a longer incubation period to develop. Additionally, this might represent a ceiling effect and compartmental convergence on saturable barrier pathways with a threshold effect of LPS ex vivo. In the perfused lung, intratracheal ECV_nic_ primarily engages the epithelial-alveolar macrophage interface with paracrine endothelial activation, whereas intravascular LPS directly stimulates the endothelium [40]. Both routes can converge on Angpt-2 release and Tie2 suppression. When these overlapping mechanisms approach a maximal leak state in the intact alveolo-capillary unit, simultaneous application may not further increase permeability, whereas in reductionist cell systems, temporal sequencing and receptor-level sensitization might reveal priming effects. Of note, fluid retention and the wet-to-dry ratio are closely related but represent different aspects of pulmonary edema, with fluid retention reflecting dynamic fluid accumulation during perfusion and the wet-to-dry ratio representing tissue water content at the experimental endpoint. Accordingly, the ex vivo results should not be interpreted as demonstrating a uniform additive effect of ECV_nic_ and LPS across all parameters. Further experiments using different exposure durations and lower LPS concentrations would be required to investigate these differences.

Considering the above results, the Angpt/Tie2 system is a potential therapeutic target in EVALI-associated endothelial injury or potential long-term effects of e-cigarette abuse on the vasculature. Therefore, we tested the novel Tie2 agonist PMC-403 in vitro and ex vivo. PMC-403 was able to activate downstream signaling of Tie2 as we and others have shown [41]. Accordingly, PMC-403 reduced susceptibility to an injurious stimulus (i.e. LPS) on endothelial barrier dysfunction in lung cells (HPMECs), although no significant effect on HUVECs was observed. We hypothesized that Tie2 activation by PMC-403 leads to a decrease in Angpt-2 in an AKT-dependent negative feedback manner as described by Daly et al. [42]. However, Angpt-2 levels remained unchanged in HUVECs, potentially explaining why no protection of endothelial barrier was seen in HUVECs. Importantly, PMC-403 reduced ECV_nic_- or LPS-induced endothelial dysfunction in the isolated lung suggesting complex regulation mechanisms which cannot be fully covered in isolated cell cultures. These findings are consistent with a post-ligand, junction-stabilizing mode of action that restores Tie2 signaling even when Angpt-2 remains elevated. Presumably, PMC-403 may have higher binding affinity to the Tie2 receptor compared to Angpt-2, thus increasing Tie2 signaling even in the presence of high Angpt-2 levels. These observations are concordant with preclinical studies demonstrating that strengthening Tie2 signaling, either by Angpt-1 mimetics or by Tie2-activating biologics that neutralize Angpt-2, reduces vascular leak and improves outcomes in systemic inflammation [32, 33, 43]. Nevertheless, off-target effects of PMC-403 without influencing the Angpt-/Tie2 system cannot be excluded.

The LPS-induced reduction of approximately 20% in our TER experiments was moderate and showed partial recovery at later time points. Similar magnitudes and kinetics have, however, been reported in ECIS-based studies, including a 20–25% reduction followed by recovery in LPS-treated HUVECs [44] and a 20% reduction in human pulmonary microvascular endothelial cells [45]. Differences in endothelial cell origin, exposure conditions, culture conditions, and biological activity of individual LPS preparations may contribute to this variability. In our experiments, the LPS response was reproducible across independent measurements and corresponding barrier dysfunction was also observed in the isolated lung model.

Given the heterogeneous and evolving exposure landscape in EVALI (including nicotine content and additives), an endothelium-centered, barrier-protective approach merits further evaluation in *in vivo* models and, ultimately, clinical studies.

This study has limitations. First of all, a direct chemical analysis of ECVE was not performed in this study but has been assessed in previous studies. Thus, attribution of Angpt/Tie dysregulation to specific ECVE components is beyond the scope of this work. Furthermore, the LPS-induced TER reduction was moderate and partially reversible, limiting conclusions regarding PMC-403-mediated protection during severe and sustained barrier disruption. In addition, the absence of an ECVE_nic_-alone TER control limited assessment of the independent ECVE_nic_ effect and of a potential additive or synergistic interaction with LPS. In addition, Angpt-2 transcription was assessed at a single time point, precluding assessment of potentially different temporal transcriptional responses to ECVE_nic_ and LPS. Most of our in vitro mechanistic data rely on HUVECs, which differ from HPMECs in phenotype and baseline Tie2 regulation. However, barrier protection by PMC-403 was demonstrated only in HPMECs. Generalizability is limited by lack of further exploration in systemic immune responses and interactions in the employed model. For this an *in vivo* model is required. Additionally, we were not able to identify the full mechanism of the anti-permeability effect of the Tie2 agonist PMC-403. Lastly, Angpt-2 cleavage products (25 and 50 kDa) have recently been shown to have differential effects on either agonizing or antagonizing Tie2 [46]. Further addressing these gaps will be important for translation of a possible therapeutic agent for EVALI. Nevertheless, this study provides the first mechanistical insight into e-cigarette vapor induced pathophysiology of endothelial damage.

## Conclusions

Our data show that e-cigarette vapor increases endothelial permeability and the susceptibility to LPS-induced endothelial damage via Tie2 signaling and most likely an increase of Angpt-2 levels. We identified the novel Tie2 agonist PMC-403 as a potential therapeutic agent for EVALI as it is able to reduce inflammation-mediated permeability in vitro and ex vivo.

## Supplementary Information

Supplementary Material 1: Supplemental Data 1: E-cigarette vapor application in isolated ventilated and perfused mouse lungs. To isolate the lung/heart block, mice were deeply anesthetized with ketamine (100 mg/kg body weight, i.p.) and xylazine (20 mg/kg body weight, i.p.), following anticoagulation with heparin (2,500 U/kg body weight). A tracheostomy was performed for intubation, and the mice were ventilated with a normoxic gas mixture (21% O₂, 5.3% CO₂, balanced with N₂) using a piston pump (Minivent Type 845, Hugo Sachs Elektronik, March-Hugstetten, Germany). Ventilation settings included a tidal volume of 10 μl/g body weight, a respiratory rate of 90 breaths/min (60 breaths/min during direct e-cigarette vapor inhalation), and a positive end-expiratory pressure (PEEP) of 3 cm H₂O. While maintaining ventilation, the chest was opened, and the block of lung and heart was dissected. Perfusion was initiated via a catheter inserted into the pulmonary artery using a peristaltic pump (ISM834A V2.10, Ismatec, Wertheim, Germany). The perfusate consisted of Krebs-Henseleit buffer [120 mM NaCl, 4.3 mM KCl, 1.1 mM KH₂PO₄, 2.4 mM CaCl₂, 1.3 mM MgCl₂, 13.3 mM glucose, and 5% (w/v) hydroxyethyl amylopectin; molecular weight 200,000 Da]. Following removal of the lung-heart block from the thoracic cavity, the preparation was freely suspended from a scale to monitor weight changes. A catheter was inserted into the left atrium to collect the outflow perfusate. The outflow pressure was maintained at 2 cm H₂O. Pulmonary arterial pressure, venous pressure, ventilation pressure, and lung weight were continuously monitored throughout the experiment. Nicotine-containing e-cigarette vapor (ECV_nic_) was generated using the “eVic Primo Mini” battery pack (Joyetech®) and the “Eleaf Melo 3” clearomizer (0.5 Ω, 30 W; Riccardo®, Riccardo Retail GmbH, Neubrandenburg, Germany). Vapor was drawn into a perfusor syringe via a vacuum pump using normoxic air. The puffing protocol involved four 5-second puffs (20 seconds total). ECV_nic_ was applied to the isolated lung via the inspiratory tube using a syringe driver, mimicking normal ventilation. During each application, the expiratory tube was closed until the target inspiratory volume was reached, then reopened to release the vapor. This process lasted approximately 2 seconds and was repeated 150 times over a 5-minute period. In total, seven ECV_nic_ application cycles were performed at 10-minute intervals. Normoxic ventilation was maintained throughout. Control experiments followed the same protocol but used normoxic gas without ECV_nic_. 6 µg/ml LPS, 10 µg/ml of the Tie2-agonist PMC-403, or their combination was added to the perfusate 5 minutes before ECV_nic_ inhalation. After ECV_nic_ inhalation, transvascular fluid filtration (endothelial permeability) was assessed under hydrostatic stress by increasing venous pressure from 2 to 12 cm H₂O for 8 minutes (“pressure challenge”), using the capillary filtration coefficient (K_fc_) as previously described [23]. Two pressure challenges were performed before the start of PMC-403 application to confirm the quality and stability of the lung preparation. The entire experiment lasted approximately 4 hours. To assess edema formation, net lung weight gain following a hydrostatic challenge was measured as an indicator of fluid retention. Lungs were suspended from a force transducer, and weight gain was calculated as the difference between the baseline and 5 minutes after each pressure challenge. Additionally, wet-to-dry lung weight ratios were determined using the left lungs. Wet weight was recorded immediately after the experiment, and dry weight was measured after a 48-hour drying period at 65°C.

Supplementary Material 2: Figure S1: ECVE does not influence adhesion molecules at baseline. HUVECs were stimulated with either ECVE_base_ (*n*=4) or ECVE_nic_ (*n*=5) at different concentrations for 24 hours and (A) Tie2, (B) ICAM and (C) VCAM mRNA expression were measured by qPCR in HUVECs after stimulation for 24 hours. Results are shown as dot plots with mean ± SD. *P*-values < 0.05 are considered significant. Abbreviations: ECVE – e-cigarette vapor extract, HUVECs – human umbilical vein endothelial cells, ICAM - Intercellular adhesion molecule, SD – standard deviation, VCAM - Vascular cell adhesion molecule.

Supplementary Material 3: Figure S2: The effect of the Tie2 agonist PMC-403 on the Angpt/Tie2 system in HUVECs. (A) HUVECs were stimulated with different concentrations of the Tie2 agonist PMC-403 for 24 hours. The Angpt-2 concentrations in the supernatant were measured by ELISA. Each group consisted of *n*=3. Data was analyzed by One-Way ANOVA with according post-hoc tests. (B) Additionally, Angpt-2 transcription was measured by qPCR after stimulation of HUVECs with PMC-403 (*n*=2) or control (*n*=3) for 8 hours and analyzed using an unpaired t-test. (C) HUVECs were incubated with 10 µg/ml PMC-403 for 1 hour and then stimulated with LPS. TER was measured by ECIS over time. Each group consisted of *n*=4. A linear mixed-effects model was used to analyze group and time differences. Results are shown as dot plots with mean ± SD. *P*-values < 0.05 are considered significant. Abbreviations: Angpt-2 - Angiopoietin-2, HUVECs – human umbilical vein endothelial cells, SD – standard deviation.

## Data Availability

The data underlying this article will be shared on reasonable request to the corresponding author.

## Abbreviations

Angpt-1: Angiopoietin–1
Angpt-2: Angiopoietin–2
ARDS: Acute respiratory distress syndrome
ECIS: Electric cell–substrate impedance sensing
ECV: E–cigarette vapor
ECVE: E–cigarette vapor extract
EVALI: E–cigarette associated acute lung injury
HPMECs: Human pulmonary microvascular endothelial cells
HUVECs: Human umbilical vein endothelial cells
ICAM: Intercellular adhesion molecule
Kfc: Capillary filtration coefficient
LPS: Lipopolysaccharide
SD: Standard deviation
TER: Transendothelial resistance
TLR: Toll–like receptor
VCAM: Vascular cell adhesion molecule
