# Supplementary material for: The Tie-2 agonist PMC-403 inhibits endothelial damage induced by e-cigarette vapor or LPS
Source: Respir Res. 2026 Sep 26;27:374. doi: 10.1186/s12931-026-03928-4 (PMC13621675; doi:10.1186/s12931-026-03928-4)
Supplement: Supplementary file 1 [file 12931_2026_3928_MOESM1_ESM.docx]

**The Tie2 agonist PMC-403 might reduce endothelial barrier dysfunction and increased susceptibility to LPS after exposure to nicotine containing e-cigarette vapor**

Thorben Pape^1,2,^*, Oleg Pak^3,^*, Theresa Graalmann^4,5^, Christine Ehlers^4^, Janik Deutscher^3^, David Spiegelberg^3^, Paul Horn^6^, Klaus Stahl^7^, Marius M. Hoeper^1,2^, Sascha David^8^, Natascha Sommer^3,#^ and Benjamin Seeliger^1,2,#^

**Affiliations**

^1^ Division of Respiratory Medicine and Infectious Diseases, Hannover Medical School and German Center for lung research (DZL), Hannover, Germany.

^2^ Biomedical Research in Endstage and Obstructive Lung Disease Hannover (BREATH), German Center for Lung Research (DZL)

^3^ Excellence Cluster Cardio-Pulmonary Institute, University of Giessen and Marburg Lung Center (UGMLC), member of the German Center for Lung Research (DZL), Institute for Lung Health (ILH), Justus Liebig University Giessen, Giessen, Germany

^4^ Center for experimental and clinical infection research, Twincore, Hannover, Germany.

^5^ Department of Rheumatology, Hannover Medical School, Hannover, Germany.

^6^ Medical Clinic for Hepatology and Gastroenterology, Charité Berlin, Campus Virchow-Klinikum and Campus Charité Mitte, Berlin, Germany.

^7^ Division of Gastroenterology, Hepatology, Infectious Diseases and Endocrinology, Hannover Medical School, Hannover, Germany.

^8^ Institute of Intensive Care Medicine, University hospital Zürich and University of Zurich, Zurich, Switzerland

* These authors contributed equally as first authors

^#^ These authors contributed equally as senior authors

Correspondence to: Benjamin Seeliger, MD & Thorben Pape, MD
Hannover Medical School, Department of Respiratory Medicine and Infectious Diseases
Carl-Neuberg-Str.1, 30625 Hannover, GERMANY; [Seeliger.Benjamin@mh-hannover.de](mailto:Seeliger.Benjamin@mh-hannover.de)

# Supplemental Data 1

### E-cigarette vapor application in isolated ventilated and perfused mouse lungs

To isolate the lung/heart block, mice were deeply anesthetized with ketamine (100 mg/kg body weight, i.p.) and xylazine (20 mg/kg body weight, i.p.), following anticoagulation with heparin (2,500 U/kg body weight). A tracheostomy was performed for intubation, and the mice were ventilated with a normoxic gas mixture (21% O₂, 5.3% CO₂, balanced with N₂) using a piston pump (Minivent Type 845, Hugo Sachs Elektronik, March-Hugstetten, Germany). Ventilation settings included a tidal volume of 10 μl/g body weight, a respiratory rate of 90 breaths/min (60 breaths/min during direct e-cigarette vapor inhalation), and a positive end-expiratory pressure (PEEP) of 3 cm H₂O. While maintaining ventilation, the chest was opened, and the block of lung and heart was dissected. Perfusion was initiated via a catheter inserted into the pulmonary artery using a peristaltic pump (ISM834A V2.10, Ismatec, Wertheim, Germany). The perfusate consisted of Krebs-Henseleit buffer [120 mM NaCl, 4.3 mM KCl, 1.1 mM KH₂PO₄, 2.4 mM CaCl₂, 1.3 mM MgCl₂, 13.3 mM glucose, and 5% (w/v) hydroxyethyl amylopectin; molecular weight 200,000 Da].

Following removal of the lung-heart block from the thoracic cavity, the preparation was freely suspended from a scale to monitor weight changes. A catheter was inserted into the left atrium to collect the outflow perfusate. The outflow pressure was maintained at 2 cm H₂O. Pulmonary arterial pressure, venous pressure, ventilation pressure, and lung weight were continuously monitored throughout the experiment.

Nicotine-containing e-cigarette vapor (ECV_nic_) was generated using the “eVic Primo Mini” battery pack (Joyetech®) and the “Eleaf Melo 3” clearomizer (0.5 Ω, 30 W; Riccardo®, Riccardo Retail GmbH, Neubrandenburg, Germany). Vapor was drawn into a perfusor syringe via a vacuum pump using normoxic air. The puffing protocol involved four 5-second puffs (20 seconds total). ECV_nic_ was applied to the isolated lung via the inspiratory tube using a syringe driver, mimicking normal ventilation. During each application, the expiratory tube was closed until the target inspiratory volume was reached, then reopened to release the vapor. This process lasted approximately 2 seconds and was repeated 150 times over a 5-minute period. In total, seven ECV_nic_ application cycles were performed at 10-minute intervals. Normoxic ventilation was maintained throughout. Control experiments followed the same protocol but used normoxic gas without ECV_nic_.

6 µg/ml LPS, 10 µg/ml of the Tie2-agonist PMC-403, or their combination was added to the perfusate 5 minutes before ECV_nic_ inhalation.

After ECV_nic_ inhalation, transvascular fluid filtration (endothelial permeability) was assessed under hydrostatic stress by increasing venous pressure from 2 to 12 cm H₂O for 8 minutes (“pressure challenge”), using the capillary filtration coefficient (Kfc) as previously described [1]. Two pressure challenges were performed before the start of PMC-403 application to confirm the quality and stability of the lung preparation. The entire experiment lasted approximately 4 hours.

To assess edema formation, net lung weight gain following a hydrostatic challenge was measured as an indicator of fluid retention. Lungs were suspended from a force transducer, and weight gain was calculated as the difference between the baseline and 5 minutes after each pressure challenge. Additionally, wet-to-dry lung weight ratios were determined using the left lungs. Wet weight was recorded immediately after the experiment, and dry weight was measured after a 48-hour drying period at 65°C.

# References

1.	Seeger W, Walmrath D, Grimminger F, Rosseau S, Schutte H, Kramer HJ, Ermert L, Kiss L: **Adult respiratory distress syndrome: model systems using isolated perfused rabbit lungs.** *Methods Enzymol* 1994, **233:**549-584.
